# Testosterone Replacement Therapy as a Foundation for Body Composition Remodeling: Synergistic Roles of Resistance Training and Protein Intake

**DOI:** 10.7759/cureus.109931

**Published:** 2026-05-30

**Authors:** Luis M Canal de Velasco, José Emiliano González Flores, Gabriel Kraus Fischer, Mariana de la Vega, Jose Luis Morales Arteaga, Andres Bello Margalef, Santiago Segón Mora, Alan Abruch-Fleisher, Alberto Azcona Cervera

**Affiliations:** 1 School of Medicine and Health Sciences, Panamerican University, Mexico City, MEX; 2 School of Medicine and Health Sciences, Tecnológico de Monterrey Mexico City Campus, Mexico City, MEX; 3 Master of Public Health Program, Anahuac University North Campus, Mexico City, MEX; 4 Department of Bariatric Surgery, ABC Medical Center, Mexico City, MEX; 5 Department of Dermatopathology, Dr. Ladislao de la Pascua Dermatological Center, Mexico City, MEX; 6 Department of Plastic and Reconstructive Surgery, Restora Surgical México, Mexico City, MEX; 7 School of Health Sciences, La Salle University Mexico City Campus, Mexico City, MEX; 8 Institute of Psychology, Psychiatry and Neuroscience, King's College London, London, GBR; 9 Department of Traumatology and Orthopedics, ABC Hospital, Santa Fe Campus, Mexico City, MEX

**Keywords:** body composition, lean body mass, metabolic health, protein intake, resistance training, testosterone replacement therapy

## Abstract

Testosterone plays a central role in the regulation of body composition, skeletal muscle metabolism, and metabolic health in men. Testosterone deficiency is frequently associated with increased adiposity, reduced lean body mass, impaired physical performance, and adverse metabolic profiles, contributing to the development of sarcopenia and cardiometabolic disease. Testosterone replacement therapy (TRT) has emerged as an effective intervention to restore physiological androgen levels and improve body composition by promoting increases in lean mass and reductions in fat mass.

This review proposes a conceptual framework in which TRT functions as the biological foundation upon which lifestyle interventions exert amplified anabolic effects. Mechanistic and clinical data demonstrate that TRT enhances muscle protein synthesis, satellite cell activation, and mitochondrial function, thereby supporting both the quantity and quality of skeletal muscle. When combined with resistance exercise, TRT amplifies hypertrophic responses and functional performance, while adequate protein intake provides the necessary substrates to sustain muscle remodeling and preserve fat-free mass.

This integrated framework highlights the limitations of relying solely on body weight as a clinical metric and underscores the importance of evaluating body composition changes in the context of metabolic health. When appropriately prescribed and combined with targeted lifestyle interventions, TRT may represent a comprehensive strategy for improving musculoskeletal integrity, enhancing metabolic function, and reducing the burden of hypogonadism-related complications. Further research is warranted to refine patient selection, optimize treatment protocols, and clarify long-term clinical outcomes.

## Introduction and background

Obesity and unfavorable body composition represent major global health challenges, contributing substantially to the development of metabolic disorders, cardiovascular disease, and reduced physical function. Beyond total body weight, the relative proportions of lean body mass and adipose tissue play a critical role in determining metabolic health and long-term clinical outcomes. Skeletal muscle is a key metabolic organ involved in glucose metabolism, insulin sensitivity, and energy expenditure, and reductions in muscle mass have been associated with increased cardiometabolic risk and functional decline [1,2,3]. Consequently, strategies aimed at improving body composition have become a central focus in metabolic and endocrine research.

Testosterone is a fundamental endocrine regulator of body composition in men. Physiological testosterone levels contribute to the maintenance of skeletal muscle mass, regulation of adipose tissue distribution, preservation of metabolic homeostasis, and optimization of protein-mediated anabolic processes [4]. In contrast, testosterone deficiency is frequently associated with increased visceral adiposity, reduced lean body mass, decreased physical performance, and a higher prevalence of metabolic disorders, including insulin resistance and type 2 diabetes mellitus [5]. In this context, testosterone replacement therapy (TRT) has emerged as an important therapeutic intervention for appropriately selected men with hypogonadism, with potential benefits extending beyond symptom control toward musculoskeletal and metabolic health [6].

Despite growing evidence supporting the effects of TRT on body composition, the magnitude of these changes may be strongly influenced by modifiable lifestyle factors. Resistance training represents a potent mechanical stimulus for skeletal muscle hypertrophy, while adequate dietary protein intake provides the essential substrates required for muscle protein synthesis [4]. However, the combined interaction between hormonal restoration, resistance exercise, and nutritional optimization in shaping body composition has not been consistently emphasized in the literature. Understanding how these factors interact may provide a more comprehensive framework for maximizing the anabolic and metabolic benefits associated with testosterone therapy.

The aim of this narrative review is to examine the role of testosterone replacement therapy in body composition remodeling among men with hypogonadism and to explore how resistance training and dietary protein intake may enhance these effects. By integrating evidence from endocrine physiology, clinical trials, and metabolic research, this review proposes a physiologically reasoned conceptual model in which TRT provides the endocrine context for anabolic adaptation, while resistance exercise and adequate protein intake act as complementary interventions that may support muscle remodeling and metabolic health. Importantly, this model should be interpreted as an integrative framework derived from parallel lines of evidence rather than as a directly validated combined intervention protocol.

## Review

Methods

Study Design

A semi-systematic narrative review methodology was employed to synthesize the current evidence regarding the role of TRT in improving body composition and metabolic health in men with low serum testosterone levels. This approach was selected to integrate findings from experimental studies, clinical trials, and observational research examining the effects of TRT on lean body mass, fat mass distribution, and metabolic parameters. Particular attention was given to evidence exploring the synergistic interactions between hormonal restoration, resistance training, and dietary protein intake as complementary strategies that may enhance anabolic adaptations and promote favorable body composition remodeling. Given the heterogeneity across study populations, intervention protocols, lifestyle factors, and methods used to assess body composition, a formal systematic review or quantitative meta-analysis was not feasible; therefore, a narrative synthesis was considered the most appropriate methodological framework to contextualize the available evidence.

The review integrates mechanistic, clinical, and translational evidence to construct a physiologically coherent framework while maintaining transparency in search strategy and study selection.

Literature Search Strategy

A comprehensive and structured literature search was conducted across PubMed/Medical Literature Analysis and Retrieval System Online (MEDLINE), Excerpta Medica database (Embase), Scopus, Web of Science, and the Cochrane Library to identify relevant publications addressing the effects of testosterone and TRT on body composition, skeletal muscle physiology, and metabolic health. Particular emphasis was placed on studies evaluating changes in lean body mass, fat mass distribution, and metabolic outcomes in men with low serum testosterone levels, as well as evidence exploring the interaction between TRT, resistance training, and dietary protein intake in promoting favorable body composition remodeling.

The search period extended from January 1990 through March 2025 in order to capture foundational mechanistic research on androgen signaling and skeletal muscle metabolism, as well as contemporary clinical studies evaluating the effects of TRT on body composition and metabolic parameters.

The search strategy combined Medical Subject Headings (MeSH) and free-text terms, including “testosterone replacement therapy,” “TRT,” “testosterone deficiency,” “hypogonadism,” “androgens,” “lean mass,” “fat-free mass,” “skeletal muscle,” “body composition,” “resistance training,” “protein intake,” “muscle hypertrophy,” “metabolic health,” and “sarcopenia.” Boolean operators (AND/OR) were applied as appropriate to refine the search strategy.

Reference lists of key clinical trials, meta-analyses, and review articles were manually screened to ensure completeness, and citation tracking was performed to identify additional relevant studies. Only peer-reviewed articles published in English were considered eligible for inclusion. Priority was given to randomized clinical trials, meta-analyses, and large observational studies.

Study Selection and Eligibility Criteria

Eligible publications included randomized controlled trials, prospective and retrospective observational studies, mechanistic and translational investigations, meta-analyses, and high-quality narrative reviews examining: (1) the effects of testosterone deficiency and TRT on body composition, skeletal muscle physiology, and fat distribution; (2) the impact of resistance training on skeletal muscle hypertrophy and metabolic adaptations; and (3) the role of dietary protein intake in supporting muscle protein synthesis and preserving lean body mass. Particular emphasis was placed on studies evaluating changes in lean body mass, fat mass, muscle strength, and metabolic parameters in adult men with low serum testosterone levels.

Studies exclusively evaluating supraphysiologic androgen administration, anabolic steroid misuse, or performance enhancement in eugonadal athletes were excluded. Editorials, opinion pieces, conference abstracts, and non-peer-reviewed sources were also excluded to ensure methodological rigor and the inclusion of high-quality evidence.

Because TRT is primarily indicated for men with testosterone deficiency and most clinical outcome data are derived from male endocrine reference ranges, the evidence base included in this review was predominantly limited to adult male populations. Studies involving female cohorts were considered only when relevant to mechanistic insights into androgen signaling and skeletal muscle physiology, but they were not used to extrapolate clinical conclusions regarding TRT.

Titles and abstracts were independently screened by two reviewers. Full-text evaluation was subsequently performed for studies meeting preliminary eligibility criteria. Any disagreements regarding study inclusion were resolved through discussion and consensus.

PRISMA-Informed Reporting

To enhance methodological transparency and reproducibility, elements of systematic review reporting were incorporated. The initial database search yielded 412 records. After removal of 108 duplicates, 304 records were screened at the title and abstract level. Of these, 229 studies were excluded due to irrelevance to testosterone-related body composition outcomes, lack of clinically relevant testosterone or TRT data, or focus on non-clinical anabolic steroid misuse.

Seventy-five full-text articles were subsequently assessed for eligibility. Following detailed evaluation, 55 articles were excluded for not meeting predefined inclusion criteria, including insufficient body composition data, lack of relevance to testosterone replacement therapy, absence of resistance training or nutritional considerations when applicable, or lack of clinically interpretable outcomes related to lean mass or fat mass.

**Figure 1:**
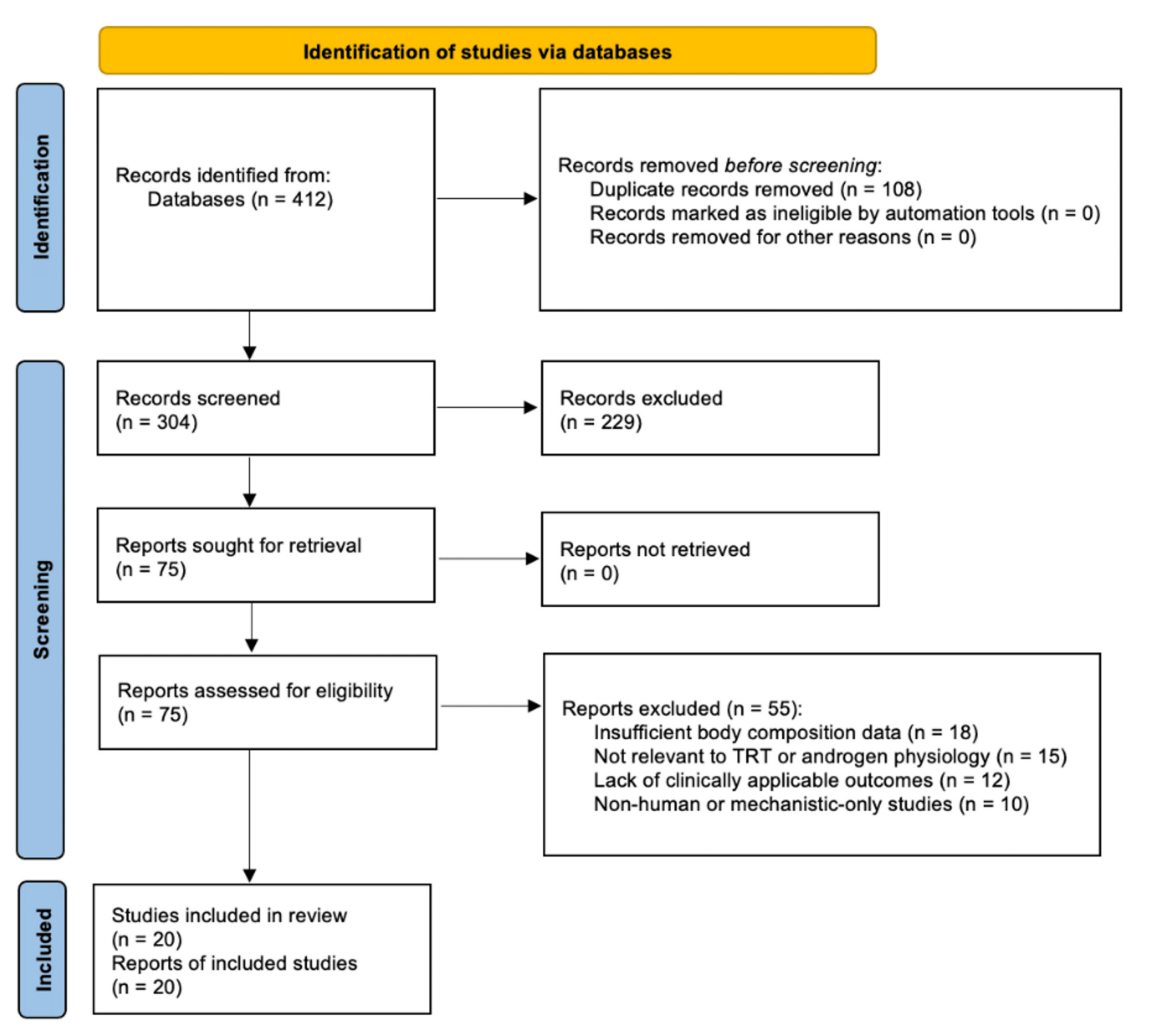
PRISMA 2020–inspired flow diagram of study selection Flow diagram illustrating the study selection process for this narrative review. A total of 412 records were identified through database searching. After removal of 108 duplicate records, 304 studies were screened based on title and abstract, of which 229 were excluded due to lack of relevance to testosterone-related body composition outcomes or methodological limitations. Seventy-five full-text articles were assessed for eligibility, and 55 were excluded for predefined reasons, including insufficient body composition data, lack of relevance to testosterone replacement therapy or androgen physiology, absence of clinically applicable outcomes, or non-human/mechanistic-only designs. Ultimately, 20 studies were included in the final qualitative synthesis.

A total of 20 studies were ultimately included in the final qualitative synthesis. The study selection process is summarized in a PRISMA 2020-inspired flow diagram (Figure 1) illustrating records identified, screened, assessed for eligibility, and included in the narrative synthesis. This PRISMA-informed framework was used to enhance reporting clarity and methodological transparency rather than to indicate a fully systematic review methodology.

Results

Physiological Role of Testosterone in Body Composition Regulation

Testosterone is a key endocrine regulator of body composition, skeletal muscle metabolism, and fat distribution in men. Physiologically, testosterone exerts anabolic effects on skeletal muscle by activating androgen receptors within myocytes, stimulating satellite cell proliferation, and enhancing muscle protein synthesis, processes that collectively contribute to the maintenance and growth of lean body mass [7,8,9]. These mechanisms also involve modulation of intracellular signaling pathways related to mitochondrial function and cellular metabolism, which further support muscle performance and energy utilization [9,10]. In contrast, declining testosterone levels are associated with reductions in lean body mass, increased adiposity, and impaired metabolic homeostasis, highlighting the central role of androgens in maintaining a healthy body composition profile [11,12].

Low serum testosterone level represents a clinical condition in which inadequate testosterone production leads to a constellation of metabolic and musculoskeletal alterations. Men with low circulating testosterone frequently exhibit increased visceral adiposity, reduced muscle mass, decreased physical performance, and a higher prevalence of metabolic disorders such as insulin resistance and type 2 diabetes mellitus [13,14]. These alterations contribute to unfavorable shifts in body composition characterized by the simultaneous accumulation of fat mass and loss of skeletal muscle tissue. In aging populations, this imbalance may progress toward conditions such as sarcopenia or sarcopenic obesity, which are associated with increased morbidity, reduced functional capacity, and poorer metabolic outcomes [15,16].

The interaction between hormonal status and body composition becomes particularly relevant in the context of modern obesity management strategies. While pharmacological weight-loss therapies, including glucagon-like peptide-1 (GLP-1) receptor agonists, have demonstrated substantial reductions in body weight, emerging evidence suggests that part of this weight loss may include reductions in lean body mass alongside fat mass [1,2,6]. Loss of skeletal muscle during weight reduction may negatively affect metabolic health, basal metabolic rate, and long-term weight maintenance. Consequently, strategies that preserve or enhance lean mass while reducing adiposity have gained increasing attention in metabolic medicine.

Within this physiological framework, testosterone has been increasingly recognized as a metabolic hormone that influences not only reproductive function but also systemic energy balance and body composition regulation [10]. Experimental and clinical studies have demonstrated that adequate testosterone levels contribute to the maintenance of skeletal muscle mass, improved muscle function, and favorable metabolic profiles, emphasizing the importance of androgen signaling in the preservation of lean tissue and the prevention of excess fat accumulation [17,18,19]. These findings provide the biological foundation for therapeutic approaches aimed at restoring testosterone levels in men with low testosterone serum levels as a means of improving body composition and metabolic health.

Effects of TRT on Lean Mass and Fat Mass

Restoration of physiological testosterone levels through TRT has been associated with clinically relevant changes in body composition among men with hypogonadism. Rather than producing a uniform reduction in total body weight, TRT appears to modify the relative distribution of lean and adipose tissue through androgen receptor-mediated effects on skeletal muscle, adipose tissue metabolism, and systemic energy balance. These effects provide a biological rationale for evaluating treatment response using body composition outcomes rather than body weight alone.

Controlled clinical studies have provided strong evidence supporting these anabolic effects. Experimental models have shown that testosterone administration increases muscle fiber size, improves muscle strength, and enhances functional capacity, particularly in aging men with reduced endogenous testosterone production [20,21]. In randomized clinical trials, testosterone therapy has been associated with measurable increases in lean mass and improvements in physical performance, confirming the physiological role of testosterone in maintaining musculoskeletal integrity [22,23]. These findings are particularly relevant in older populations, where declining androgen levels often coincide with progressive loss of muscle mass and strength.

From a clinical perspective, the effects of TRT on body composition should be interpreted in relation to treatment formulation, dose, route of administration, frequency, and duration of therapy. Injectable testosterone regimens commonly include testosterone cypionate or enanthate administered intramuscularly, with the FDA-recommended dosing range for testosterone cypionate being 50-400 mg every two to four weeks as a deep intramuscular injection [24]. Treatment duration across clinical studies varies substantially, ranging from short-term interventions of approximately 12-16 weeks to longer protocols extending six to 12 months or more, which may partly explain differences in the magnitude of body composition responses. Reported changes generally include increases in fat-free or lean mass and reductions in fat mass, although the absolute and relative magnitude vary according to baseline testosterone status, age, adiposity, training exposure, nutritional intake, and adherence. Across selected clinical studies, reported lean mass gains have ranged from approximately 1-3 kg, while fat mass reductions have ranged from approximately 1-4 kg, corresponding in some cohorts to relative changes of roughly 2%-5% in lean mass and 3%-10% in fat mass. These findings support the concept that TRT modifies body composition most meaningfully when treatment exposure is adequate and when outcomes are evaluated beyond total body weight alone.

Beyond its effects on skeletal muscle, TRT has also demonstrated important metabolic benefits through its influence on adipose tissue distribution. Several studies have reported reductions in total fat mass and visceral fat following testosterone therapy, suggesting that restoration of normal androgen levels may promote a more favorable metabolic profile [25,26]. This reduction in adiposity may partly result from increased energy expenditure, improved insulin sensitivity, and enhanced mitochondrial activity in skeletal muscle and adipose tissue [27]. Long-term observational data further suggest that sustained testosterone therapy may contribute to progressive weight reduction and improvements in parameters associated with metabolic syndrome.

Meta-analyses examining testosterone supplementation have confirmed these observations, demonstrating consistent increases in fat-free mass and reductions in fat mass among men receiving TRT [16]. Importantly, these changes in body composition are not always reflected by dramatic changes in total body weight, as increases in lean tissue may offset reductions in adipose tissue. Consequently, evaluating the metabolic impact of TRT requires assessment of body composition rather than reliance on weight alone.

Overall, the available evidence supports TRT as a body composition-modifying intervention in appropriately selected men with hypogonadism. Its clinical relevance lies not only in restoring androgen levels but also in creating a physiological environment in which musculoskeletal, metabolic, and lifestyle-directed interventions may exert greater benefit.

Synergistic Effects of TRT and Resistance Training

While TRT alone can induce meaningful improvements in body composition, the anabolic effects of testosterone are significantly amplified when combined with resistance training. Resistance exercise is widely recognized as one of the most potent physiological stimuli for skeletal muscle hypertrophy, primarily through mechanical loading that activates intracellular pathways involved in muscle protein synthesis, including the mechanistic target of rapamycin (mTOR) signaling cascade. When adequate androgen levels are present, skeletal muscle appears to respond more efficiently to these anabolic stimuli, resulting in greater gains in muscle mass, strength, and functional capacity.

Testosterone enhances the adaptive response of skeletal muscle to mechanical stress through multiple biological mechanisms. Activation of androgen receptors in muscle tissue increases satellite cell proliferation and differentiation, processes that are essential for muscle fiber repair and hypertrophic growth following resistance exercise [10,11]. In addition, testosterone influences mitochondrial function and energy metabolism, promoting greater muscle efficiency and improved muscular performance [27]. These mechanisms collectively contribute to a more robust anabolic environment that facilitates muscle remodeling during periods of physical training.

Clinical and experimental studies support the concept that testosterone status modulates the magnitude of exercise-induced muscular adaptations. Individuals with normalized testosterone levels often experience greater improvements in muscle strength, muscle cross-sectional area, and physical performance when participating in structured resistance training programs compared with those with untreated low testosterone serum levels. Restoration of physiological androgen levels may therefore enhance the body's capacity to respond to mechanical stimuli and accelerate the process of muscle hypertrophy.

Beyond its direct effects on muscle tissue, the combination of TRT and resistance training may also influence overall metabolic efficiency. Increased skeletal muscle mass is associated with higher resting metabolic rate and improved glucose utilization, both of which contribute to more favorable metabolic profiles. Furthermore, resistance exercise promotes the mobilization and oxidation of fatty acids, which may enhance the fat-reducing effects observed with testosterone therapy. As a result, the interaction between hormonal restoration and structured resistance training represents a synergistic strategy for improving body composition by simultaneously increasing lean mass and reducing adiposity.

Taken together, these findings suggest that TRT provides the hormonal foundation for anabolic balance, while resistance training acts as a critical mechanical stimulus that maximizes the physiological potential for muscle hypertrophy. This synergistic interaction highlights the importance of integrating lifestyle interventions with hormonal therapy in order to optimize changes in body composition and improve long-term metabolic outcomes.

Role of Dietary Protein Intake in Maximizing Anabolic Effects

In addition to hormonal restoration and mechanical stimulation through resistance training, adequate nutritional support is essential to fully optimize improvements in body composition. Among nutritional factors, dietary protein intake plays a central role in supporting skeletal muscle remodeling and sustaining the anabolic effects induced by testosterone and exercise. Protein provides the essential amino acids required for muscle protein synthesis and contributes to the preservation and expansion of fat-free mass during metabolic interventions.

Muscle hypertrophy and repair require a continuous supply of amino acids to support the synthesis of new contractile proteins. Experimental studies have demonstrated that higher protein intake enhances muscle protein synthesis and facilitates greater retention of lean body mass, particularly during periods of weight loss or metabolic stress [4]. In the context of resistance training, sufficient dietary protein has been shown to amplify the anabolic signaling triggered by mechanical loading, thereby promoting greater increases in skeletal muscle mass and strength.

The interaction between dietary protein intake and testosterone-mediated anabolic signaling further supports the importance of nutritional optimization. Testosterone stimulates muscle protein synthesis by increasing the activation of androgen receptors and enhancing the efficiency of intracellular pathways involved in muscle growth. When adequate protein intake is available, these anabolic processes can proceed more effectively, allowing the organism to translate hormonal and mechanical stimuli into measurable increases in lean body mass.

Importantly, maintaining adequate protein intake may also help mitigate the loss of lean tissue that frequently occurs during weight reduction strategies. Pharmacologic weight-loss interventions, including GLP-1 receptor agonists, have demonstrated substantial reductions in body weight; however, part of this reduction may involve decreases in lean body mass alongside fat mass [1,2,6]. Adequate protein intake, particularly when combined with resistance training, can help preserve skeletal muscle while promoting preferential reductions in adipose tissue.

Consequently, the integration of TRT, resistance training, and optimized protein intake represents a comprehensive physiological approach to body composition remodeling. Within this framework, TRT provides the hormonal environment that facilitates anabolic signaling, resistance exercise supplies the mechanical stimulus necessary for muscle hypertrophy, and dietary protein delivers the metabolic substrate required for muscle repair and growth. Together, these interventions create a synergistic strategy capable of promoting increases in lean body mass while simultaneously reducing adiposity.

Integrated Model of Body Composition Remodeling

The available evidence supports an integrated framework in which hormonal restoration, mechanical stimulation, and nutritional support interact to shape body composition outcomes in men with hypogonadism. Within this model, TRT restores the endocrine context required for anabolic signaling, while resistance training and adequate protein intake determine how effectively this hormonal environment is translated into structural and functional adaptations.

However, hormonal restoration alone does not fully exploit the physiological potential for anabolic adaptation. Resistance training introduces a powerful mechanical stimulus that further activates intracellular pathways involved in muscle hypertrophy, including increased muscle protein synthesis and satellite cell activity. When adequate testosterone levels are present, skeletal muscle appears to respond more efficiently to this mechanical stress, resulting in greater increases in muscle mass and functional strength. The combination of TRT and resistance training, therefore, represents a synergistic strategy that amplifies the adaptive response of skeletal muscle and enhances improvements in body composition.

The addition of adequate dietary protein intake provides the metabolic substrate necessary to sustain these anabolic processes. Protein supplies the essential amino acids required for muscle protein synthesis, allowing the organism to effectively translate hormonal and mechanical stimuli into structural changes in muscle tissue. When sufficient dietary protein is consumed, the combined effects of TRT and resistance exercise may lead to more pronounced gains in lean body mass while promoting preferential reductions in adipose tissue.

Taken together, this integrated model suggests that optimal body composition remodeling is achieved through the interaction of three key components: hormonal restoration, mechanical stimulation, and nutritional support. TRT establishes the endocrine environment necessary for anabolic signaling, resistance training provides the stimulus for muscular adaptation, and dietary protein intake ensures the availability of substrates required for muscle growth and repair. Through this multidimensional approach, improvements in body composition extend beyond simple changes in body weight and instead reflect a redistribution of body tissues characterized by increased skeletal muscle mass and reduced fat accumulation.

Implications for Metabolic Health

Improvements in body composition extend beyond musculoskeletal benefits and have important implications for overall metabolic health. Skeletal muscle is a major metabolic organ that plays a central role in glucose utilization, insulin sensitivity, and energy expenditure. Consequently, increases in lean body mass and reductions in adipose tissue may significantly influence metabolic homeostasis and reduce the risk of cardiometabolic disease.

Testosterone deficiency has been consistently associated with adverse metabolic profiles, including increased visceral adiposity, insulin resistance, dyslipidemia, and a higher prevalence of type 2 diabetes mellitus. These alterations contribute to the development of metabolic syndrome and are frequently observed in men with untreated hypogonadism. Restoration of physiological testosterone levels through TRT has been shown to improve several metabolic parameters, including reductions in visceral fat, improvements in insulin sensitivity, and favorable changes in lipid metabolism [17,22,23]. These effects are likely mediated by the combined influence of testosterone on skeletal muscle metabolism, adipose tissue distribution, and systemic inflammatory pathways.

The increase in skeletal muscle mass induced by TRT and resistance training may further enhance metabolic efficiency. Greater muscle mass is associated with increased basal metabolic rate and improved glucose uptake, both of which contribute to better glycemic control and long-term metabolic stability. In parallel, reductions in visceral adiposity may decrease pro-inflammatory cytokine activity and improve cardiometabolic risk profiles [25,26].

From a clinical perspective, these findings highlight the importance of evaluating therapeutic strategies that target both hormonal and lifestyle determinants of body composition. Interventions that simultaneously increase lean body mass and reduce fat mass may provide meaningful metabolic benefits, particularly in men with hypogonadism who are at increased risk for obesity-related complications [27]. Within this context, testosterone replacement therapy, when appropriately prescribed and combined with resistance exercise and adequate nutritional support, may represent a comprehensive strategy for improving body composition and promoting better metabolic health outcomes.

Discussion

The present narrative review integrates current evidence to propose a conceptual framework in which TRT serves as the biological foundation for body composition remodeling, while resistance training and adequate protein intake act as synergistic amplifiers of anabolic and metabolic adaptations. This multidimensional perspective is particularly relevant in the context of hypogonadism, where hormonal deficiency disrupts the balance between lean mass and adiposity, ultimately contributing to adverse metabolic outcomes.

At a mechanistic level, the effects of testosterone on skeletal muscle extend beyond simple anabolic signaling. Testosterone has been shown to influence mitochondrial biogenesis and energy metabolism, thereby enhancing muscular efficiency and contributing to favorable changes in body composition [28]. These findings suggest that TRT not only promotes increases in lean mass but also improves the qualitative aspects of skeletal muscle, including metabolic activity and functional capacity. This is particularly important given that muscle quality, rather than quantity alone, has emerged as a key determinant of metabolic health and physical performance [29]. In parallel, molecular and physiological studies have demonstrated that testosterone enhances muscle protein synthesis, satellite cell activation, and neuromuscular function, reinforcing its central role in musculoskeletal integrity [30].

In addition to its direct androgen receptor-mediated actions, testosterone may interact with the growth hormone/insulin-like growth factor-1 (GH/IGF-1) axis to support skeletal muscle remodeling. The GH/IGF-1 pathway plays an important role in muscle protein synthesis, satellite cell activity, and maintenance of skeletal muscle mass, and testosterone may enhance the anabolic responsiveness of this pathway during periods of hormonal restoration and mechanical loading [31,32]. Testosterone may also exert anti-catabolic effects by reducing pathways involved in muscle protein breakdown and attenuating catabolic signaling, thereby helping preserve fat-free mass under conditions of aging, metabolic stress, or caloric restriction [32,33]. Furthermore, androgen signaling may influence neuromuscular function by affecting motor neuron excitability, neuromuscular transmission, and motor unit recruitment, which may partly explain why improvements in strength or physical performance can occur even when changes in muscle mass are modest [33]. These mechanisms provide a biological basis for the combined effects of TRT, resistance training, and protein intake on both structural and functional outcomes.

From a clinical standpoint, the effects of TRT on body composition appear consistent across different age groups, although their magnitude and clinical implications may vary depending on baseline hormonal status and comorbid conditions. Prior narrative analyses have highlighted the beneficial effects of TRT on lean mass preservation, functional capacity, and overall metabolic profiles in both middle-aged and older men [34,35]. Additionally, emerging evidence suggests that TRT may exert broader systemic effects, including potential influences on cognitive and psychological domains, which may indirectly impact physical activity levels and adherence to lifestyle interventions [36]. These multidimensional effects reinforce the concept of testosterone as a systemic metabolic regulator rather than a hormone with isolated musculoskeletal actions.

Importantly, the present review emphasizes that the anabolic effects of TRT are not fully realized in isolation. Resistance training provides the mechanical stimulus necessary to activate hypertrophic pathways, while adequate dietary protein intake supplies the substrates required for muscle remodeling. This synergistic interaction aligns with existing evidence demonstrating that physical activity and nutritional factors play a critical role in preserving fat-free mass during metabolic interventions [37]. Consequently, TRT should be conceptualized not as a standalone intervention, but as part of an integrated therapeutic strategy aimed at optimizing body composition and metabolic health.

Despite these favorable effects, the use of TRT requires careful consideration of safety and appropriate patient selection. Clinical practice guidelines recommend TRT primarily for men with confirmed hypogonadism, emphasizing the importance of biochemical confirmation and symptomatology prior to initiation of therapy [35]. Concerns regarding potential adverse effects, including erythrocytosis, cardiovascular risk, and long-term safety, have been extensively debated in the literature. However, recent large-scale randomized evidence suggests that, when appropriately prescribed and monitored, TRT does not significantly increase the risk of major adverse cardiovascular events [38]. Similarly, systematic reviews and meta-analyses have reported that adverse effects are generally infrequent and manageable within a structured clinical framework [39].

Another important consideration is the heterogeneity in diagnostic criteria for hypogonadism, which may influence both patient selection and interpretation of clinical outcomes. Variability in testosterone thresholds, assay methods, and clinical definitions across different guidelines may lead to inconsistencies in treatment indications and reported outcomes [40]. This underscores the need for standardized diagnostic approaches and individualized treatment strategies based on both biochemical and clinical parameters [41,42].

The findings of this review should be interpreted in light of certain limitations. As a narrative synthesis, this work is inherently subject to selection bias and heterogeneity in study design, population characteristics, and outcome measures. Additionally, variations in TRT formulations, dosing regimens, and duration of therapy across studies may influence the magnitude of observed effects. Nevertheless, the integration of mechanistic, clinical, and lifestyle-related evidence provides a comprehensive perspective that may not be fully captured in more narrowly focused analyses. Additionally, only English-language studies were included, which may have limited the inclusion of relevant evidence published in other languages.

Despite the promising anabolic and metabolic effects associated with TRT, it is important to acknowledge that therapeutic responses are not uniform across all patients. Variability in clinical outcomes may be influenced by baseline testosterone levels, age, comorbidities, degree of metabolic dysfunction, and adherence to concomitant lifestyle interventions such as resistance training and dietary optimization. Furthermore, not all individuals experience clinically meaningful improvements in body composition, and in some cases, gains in lean mass may be modest, variable in magnitude, or reflected more prominently in functional outcomes such as strength and physical performance rather than in large structural changes in muscle mass. These considerations underscore the importance of individualized treatment strategies and realistic expectations when initiating TRT. Additionally, while current evidence supports the safety of TRT when appropriately prescribed and monitored, concerns remain regarding long-term outcomes, particularly in populations with significant cardiovascular risk or complex metabolic disease. Therefore, TRT should not be viewed as a universal or standalone solution, but rather as a component of a comprehensive, patient-centered approach that integrates hormonal therapy with evidence-based lifestyle interventions to optimize both body composition and overall health outcomes.

## Conclusions

TRT represents a physiologically grounded and clinically meaningful intervention for men with confirmed hypogonadism, with benefits that may extend beyond symptom control toward musculoskeletal and metabolic health. Its therapeutic value is greatest when hormonal restoration is integrated with resistance training and adequate dietary protein intake, allowing endocrine, mechanical, and nutritional stimuli to converge in support of functional adaptation. This framework underscores the importance of assessing treatment response through body composition, metabolic parameters, and physical function rather than body weight alone. When appropriately prescribed, individualized, and monitored, TRT may serve as one component of a comprehensive strategy to address the broader clinical burden associated with hypogonadism and related metabolic disorders.
